# Prevalence of constipation and use of laxatives, and association with risk factors among older patients during hospitalization: a cross sectional study

**DOI:** 10.1186/s12876-022-02195-z

**Published:** 2022-03-08

**Authors:** Hanne Konradsen, Veronica Lundberg, Jan Florin, Anne-Marie Boström

**Affiliations:** 1 Division of Nursing, Department of Neurobiology, Care Science and Society, Karolinska Institutet, Huddinge, Sweden; 2 Department of Clinical Medicine, Faculty of Health and Medical Sciences, University of Copenhagen, Copenhagen, Denmark; 3 Department of Gastroenterology, Herlev and Gentofte University Hospital, Borgmester Ib Juulsvej 1, 2730 Herlev, Denmark; 4 School of Education, Health and Social Studies, Dalarna University, Falun, Sweden; 5 Theme Inflammation and Aging, Karolinska University Hospital, Huddinge, Sweden; 6 Research and Development Unit, Stockholms Sjukhem, Stockholm, Sweden

**Keywords:** Constipation, Laxatives, Older, Patients, Prevalence, Risk factor

## Abstract

**Background:**

Many older patients experience constipation as a bothersome symptom with a negative impact on quality of life. During hospitalization, the focus is often on the reason for admission with the risk that other health problems are not prioritized. The aim of the study was to describe the prevalence of constipation and use of laxatives among older hospitalized patients and to investigate the associations with demographic factors, risk assessments and prescribed medications.

**Methods:**

A descriptive retrospective cross-sectional study design was used. This study enrolled patients aged 65 years or older admitted to a geriatric department. Data from electronic health records regarding constipation, demographics, risk assessments, medical diagnoses, prescribed medications and length of stay were extracted. Constipation was assessed using ICD- 10 diagnosis, documented signs and symptoms of constipation, and prescribed laxatives. Data was analyzed using descriptive and comparative analyses, including logistic regression.

**Results:**

In total, 6% of the patients had an ICD-10 diagnosis of constipation, 65% had signs and symptoms of constipation, and 60% had been prescribed laxatives. Only 5% of the patients had constipation documented according to ICD-10, signs and symptoms, and prescribed laxatives. Signs and symptoms of constipation were associated with prescribed opioids (OR = 2.254) and longer length of stay (OR = 1.063). Being prescribed laxatives was associated with longer length of stay (OR = 1.109), prescribed opioids (OR = 2.154), and older age (OR = 1.030).

**Conclusions:**

The prevalence of constipation varies depending on the methods used to identify the condition. There was a discrepancy between the documentation of constipation in relation to sign and symptoms, ICD-10 diagnosis and prescribed laxatives. The documentation of constipation was not consistent for the three methods of assessment.

## Background

Constipation is a common gastrointestinal condition, that most people have experienced at least once in their lifetime. Even though constipation is a common health condition, the existence of various definitions may partly explain why constipation is often under-diagnosed or undertreated [1].

The prevalence of constipation among older adults increases with age [2]. Constipation can lead to complications such as fecal impaction or incontinence, hemorrhoids, volvulus, or rectal prolapse [3].

Many older people experience constipation as a bothersome and distressing health condition with negative impacts on their quality of life and social activities [4]. Healthcare professionals experience that the assessment of bowel health is not prioritized, when older patients are admitted to hospital care, since the focus is on the primary symptoms related to the cause of admission [5]. A hospital stay can be challenging for older patients due to multimorbidity, in combination with changes in their environment, such as inactivity, changed eating- and drinking habits, lack of privacy and being dependent on help. This can lead to altered bowel habits and an increased risk of becoming constipated [6]. Previous research has found that constipation and laxative use are associated with higher mortality [7], and that multimorbidity might increase the likelihood of becoming constipated [8]. This can, in turn, lead to increased health care costs [9]. Healthcare professionals have also stated that they often underestimate the severity of constipation as perceived by the patient [10] and patients and physicians might have different perception of constipation [11].

The most commonly used definition of constipation is based on the Rome criteria (Mearin et al., 2016). This definition comprises a set of clinical symptoms recognized as the gold standard for constipation and is often used in clinical research [12] (Table 1). The condition constipation is described as “another functional intestinal disorder” in the International Statistical Classification of Diseases and related Health Problems (ICD-10) [13]. ICD-10 is a diagnostic classification standard used globally in clinical practice to systematically describe and communicate medical diagnoses and health-related problems. From a nursing point of view, constipation is also addressed as standardized nursing diagnoses, systematic descriptions of health conditions, e.g. the North American Nursing Diagnosis Association (NANDA) classification system. Nursing diagnosis of constipation, as an actual or a potential health problem, is defined with identified related factors or risk factors and also provides characteristics of the condition, e.g. abdominal pain, or risk factors, such as change in eating habits [14]. Further, there are several assessment scales that have been developed to support the identification of constipation, such as the Constipation Assessment Scale [15] or the Bowel Function Index [16]. In some studies the prescription of laxatives has been used as an indicator of constipation [17].

**Table 1:** Definitions of constipation by Rome IV criteria, ICD-10 (K 59) and NANDA

	Definition
Rome IV criteria	The diagnosis of constipation is met when the patient exhibits at least two of the following Rome IV Criteria: Straining for at least 1/4 (25%) of defecations Hard or lumpy stools for at least 1/4 (25%) of defecations Sensation of incomplete evacuation for at least 1/4 (25%) of defecations Sensation of abdominal obstruction/blockage for at least 1/4 (25%) of defecations Manual maneuvers to facilitate evacuation for at least 1/4 (25%) of defecations < 3 Spontaneous bowel movements per week Reference: [12]
ICD-10 (K59.0)	A condition in which stool becomes hard, dry, and difficult to pass, and bowel movements don’t happen very often. Other symptoms may include painful bowel movements and feeling bloated, oncomfortable, and sluggish A disorder characterized by irregular and infrequent or difficult evacuation of the bowels Reference: [13]
NANDA	Decrease in normal frequency of defecation, accompanied by difficult or incomplete passage of stool and/or passage of excessively hard, dry stools Reference: [14]

In a systematic review including studies worldwide, the prevalence of constipation varied between 1 and 80% among adults in different settings and using different definitions [18]. In a Swedish study, an increase in prevalence of constipation in nursing home settings was reported, from 36% in 2007 to 40% in 2013, based on healthcare professionals responses to a three item questionnaire [19]. With a definition of “no bowel movements in three days or hard stools” [20] a study found the prevalence of constipation to be 23% among nursing home residents, while 67% were prescribed laxatives for regular use. Few studies have examined the prevalence among older patients admitted to hospitals. However, one Danish study used the Constipation Assessment Scale and reported that 39% of patients indicated symptoms of constipation on admission and 43% had developed symptoms of constipation during the first three days of hospitalization [21].

Increased age is considered to be one of the most important risk factors for developing constipation, and among older people, constipation is often caused by several health conditions that sometimes interact [22]. Associated factors for constipation include physical and mobility impairments, poor appetite and impaired nutritional status [23]. Polypharmacy and specific medications, such as opioids and anticholinergics, are also associated with increased risk of constipation [19, 24]. There are also gender differences, with females being more affected than males [22]. Constipation among nursing home residents is associated with a other health risks such as risk of falls or risk of developing pressure ulcers [25].

### Rationale

The prevalence of constipation tends to be high during hospitalization with a potential negative effect on a patient’s wellbeing. The use of laxatives is a common treatment option. Few studies have been conducted on the coincidence between different assessment methods of constipation for patients during hospitalization.

## Method

The aim of this study was to describe the prevalence of constipation, the use of laxatives, and the association between constipation and demographic factors, risk assessments and prescribed medications, among older hospitalized patients.

### The following research questions were investigated

- What is the prevalence of constipation as identified in the electronic health records as either documented ICD- 10 diagnosis, described signs and symptoms of constipation, or prescribed laxatives?
- What is the prevalence of being prescribed laxatives on admission, during hospitalization and at discharge?
- What are the associations between constipation during hospitalization (dependent variable) and demographic factors, risk assessments and prescribed medications?

### Study design

This study used a descriptive, retrospective cross-sectional study design.

### Setting and sample

The study was conducted at the Department of geriatric medicine in a medium-sized hospital in a metropolitan city in Sweden, comprising six wards with 130 patient beds. The yearly admission rate is approximately 5000 patients and the patients are usually admitted to the department from acute care hospitals. The patients are generally 65 years or older (mean age 84 years) with complex medical health problems and physical restrictions as a result of debilitating illness, injury, surgery or worsening of a chronic health condition. The average length of stay is nine days.

A consecutive sampling procedure was used. Electronic health records (EHR)s for the first 40 patients discharged each month during the period January to December 2017, were identified. The inclusion criteria were adults aged 65 years or older and length of hospital stay of at least three days. Exclusion criteria were documented medical diagnosis of gastrointestinal cancer, inflammatory bowel disease (IBD), presence of an ostomy, dementia, or re-admission during the year.

### Data collection

The data extracted from the EHRs covered the information categories: demographics, risk assessments, ICD-10 diagnosis, prescribed medications, length of stay and notes written by registered nurses and physicians regarding signs and symptoms. As there is no consensus regarding how to assess and document constipation, three different data sources were used in this study in order to reach as complete a description as possible. They were as follows: data source 1—the ICD-10 diagnosis (K59.0) (Table 1); data source 2—assessments of signs and symptoms according to the definition of constipation by NANDA International [14]: “Decrease in normal frequency of defecation, accompanied by difficult or incomplete passage of stool and/or passage of excessively hard, dry stools”. In this study, signs and symptoms of constipation were present if a health professional (RN or physician) had documented the presence of elimination of dry or hard stools, difficulties with evacuation or infrequent elimination. Finally, data source 3—prescribed laxatives (ATC code A06), was used as an indirect criterion to identify constipation, a method also been used previously [17].

Demographic data were collected concerning age and sex. Risk assessments addressed three areas: nutritional status, risk for falls, and risk for developing pressure ulcers. Data concerning nutritional status were collected based on assessment by the Mini Nutritional Assessment Short Form (MNA-SF) [26]. A score of 7 points or less indicates malnutrition, 8–11 points indicates risk of malnutrition and 12–14 points indicates no risk of malnutrition. We also included Body Mass Index (BMI). Based on the GLIM criteria [27] for adults (< 70 years.), a BMI of < 20 kg/m^2^ was regarded as underweight, while a BMI of < 22 kg/m^2^ was regarded as underweight for older adults (≥ 70 years). Data were extracted regarding risk for falls described by the Downton Fall Risk Index (DFRI) [28]. A score of three or more indicates a high risk of falling. Extracted data from the EHR regarding the risk for pressure ulcers were based on assessments using the Modified Norton Scale (MNS). The maximum score is 28 and a score of 20 or lower indicates an increased risk for the development of pressure ulcers [29].

We used the Charlson Comorbidity Index (CCI) to measure comorbid conditions that predict mortality of patients based on ICD-10 diagnoses. Each category of ICD- diagnosis has an associated weight (from 1 to 6), and a higher score indicates a higher risk of mortality [30]. Data regarding prescription of opioids (ATC code N02) and anticholinergics (ATC code N04) were collected as was, data on length of stay. Based on these variables, an EHR review protocol was developed and data from the information system was extracted during 2018.

### Data analysis

The collected data were transferred to the Statistical Package of the Social Sciences version 26 for PC (IBM Corporation, Armonk, NY), and validated for correctness.

Documented signs and symptoms of constipation that were documented in the EHRs were categorized as either describing an outcome, e.g. presence of defecation or not, or being more process related, e.g. pain and abdominal distension.

The material was described using frequencies and distributions with relative proportions, mean and standard deviation (SD) or median and quartile, according to the data level. Bivariate analyses for comparisons between groups were conducted using independent *t-*test, Mann–Whitney U test and Chi-Square, depending on data level.

Logistic regression analyses were conducted for the two dependent variables, signs and symptoms of constipation and prescribed laxatives, respectively, and the independent variables related to demographics, risk assessments, comorbidity, prescribed medications, and length of stay. Some independent variables were dichotomized for the analyses, e.g. BMI, MNS and DFRI.

A *p* ≤ 0.05 was considered statistically significant.

### Ethical considerations

According to Swedish law, quality improvement such as studies with the aim to improve quality of care and patient safety are regulated in the Patient Safety Law (SOSFS 2010:659) where the Director for the department at the hospital approve the study including the data collection of anonymous/unidentified data from patient records. Thus, these studies do not require permission from the Ethical Review Authority (Ethical Review Act 2003:460). All procedures in the study were in accordance with the ethical standards of the 1964 Helsinki Declaration and its later amendments.

## Results

### Description of sample

Of 480 potential patients, 159 patient were excluded because of dementia (n = 72), gastrointestinal cancer (n = 24), short hospital stay (n = 10), IBD (n = 9), ostomy (n = 6), re-admission (23) or for miscellaneous reasons (n = 15). Thus, the final sample consisted of 321 patients, of which two-thirds were female (n = 202; 63%) (Table 2). The mean age was 84 years (SD = 8.7), with a range between 65 and 101 years. Almost a quarter were assessed to be malnourished (n = 77; 24%) and one third were underweight (n = 108; 34%). About one third were at risk of developing pressure ulcers (n = 102; 32%) and the majority of the patients were at risk of falls (n = 274; 85%). The most common medical diagnoses were cardiovascular diseases, lung diseases and diabetes. Half of the patients (50%) were prescribed opioids and nearly 14% were prescribed anticholinergics. The median length of stay on the ward was 9 days (IQR 7;12).

**Table 2:** Description of participants (N = 321) and comparisons of demographic and independent variables between patients with and without sign and symptoms of constipation and with and without prescribed laxatives

Variables	Total sample N = 321	Prescribed laxatives N = 192	Not prescribed laxatives N = 129	*P* value	Sign and symptoms of constipation N = 199	No sign and symptoms of constipation N = 122	*P* value
*Demographics*							
Age							
Mean (SD)	84 (8.7)	84 (9)	82 (9)	0.07	84 (9)	83 (9)	0.37
Sex n (%)							
Female	202 (63%)	122 (64%)	80 (62%)	0.78	123 (62%)	79 (65%)	0.60
Male	119 (37%)	70 (36%)	49 (38%)		76 (38%)	43 (35%)	
*Risk assessment*							
MNA n (%)							
No risk	88 (27%)	50 (26%)	38 (30%)	0.50	50 (25%)	38 (31%)	0.24
At risk	156 (49%)	142 (74%)	91 (71%)		149 (75%)	84 (69%)	
BMI n (%)							
Underweight	105 (33%)	58 (31%)	47 (37%)	0.25	60 (31%)	45 (37%)	0.22
Normal weight	213 (67%)	132 (69%)	81 (63%)		137 (69%)	76 (63%)	
Norton n (%)							
No risk	219 (68%)	125 (65%)	94 (73%)	0.12	127 (64%)	92 (75%)	**0.035**
At risk	101 (32%)	67 (35%)	34 (27%)		71 (36%)	30 (25%)	
Downton n (%)							
No risk	47 (15%)	25 (13%)	22 (17%)	0.32	24 (12%)	23 (19%)	0.09
At risk	274 (85%)	167 (87%)	107 (83%)		175 (88%)	99 (81%)	
*Medical diagnoses/comorbidity*							
Charlson Comorbidity Index							
Median (Q1-Q3)	2.0 (0–11)	2 (0–11)	3 (0–10)	0.17	2 (0–11)	2 (0–9)	0.63
Prescribed medications							
Laxatives during hospitalization	192 (60%)						
Yes					159 (80%)	32 (27%)	** < 0.001**
No					40 (20%)	89 (73%)	
Opioids n (%)	61 (50%)						
Yes		111 (58%)	50 (39%)	**0.001**	115 (58%)	46 (38%)	** < 0.001**
No		81 (42%)	79 (61%)		84 (42%)	76 (62%)	
Anticholinergics n (%)	44 (14%)						
Yes		30 (16%)	14 (11%)	0.223	31 (16%)	13 (11%)	0.21
No		162 (84%)	115 (89%)		168 (84%)	109 (89%)	
Length of stay							
Length of stay, days Median (Q1–Q3)	9 (7–12)	10 (8–13)	8 (7–11)	**< 0.001**	10 (8–13)	9 (7–11)	**0.004**

### Prevalence of constipation and prescribed laxatives

A total of 18 patients (6%) had received an ICD-10 diagnosis of constipation, where 2% (n = 6) were diagnosed on admission and 4% (n = 12) during the hospitalization. However, from progress notes written by RNs and physicians’ regarding signs and symptoms, 199 patients (65%) had symptoms of constipation. The signs and symptoms of constipation were most often (61%) documented as descriptions of results (presence of defecation or not) and were mostly written solely from the perspective of healthcare professionals. The description of constipation related signs and symptoms, e.g. how the patient feels, consistency of the stools, abdominal pain and nausea were less often documented.

A total of 29% (n = 93) of the patients were already prescribed laxatives at the time of admission, and the laxative prescriptions increased to 60% (n = 192) during the hospital stay. On discharge, 39% (n = 124) of the patients were prescribed laxatives. In total, 67% (n = 216) of the patients were prescribed laxatives on at least one of those three occasions. Osmotic agents were the most commonly prescribed laxative (90%), followed by stimulants (50%), enemas (18%) and bulking agents (3%).

Bivariate analysis identified that of the 199 patients having signs and symptoms of constipation, 159 were prescribed laxatives while 40 patients were not, whereof two were prescribed non-pharmacological treatments, e.g. prune juice to drink. A total of 121 patients did not have any signs and symptoms of constipation and 89 of those were not prescribed laxatives. However, 33 patients were prescribed laxatives without documented signs and symptoms of constipation, of which 12 had a prescription of laxatives already on admission to the hospital (Table 2).

**Fig. 1:**
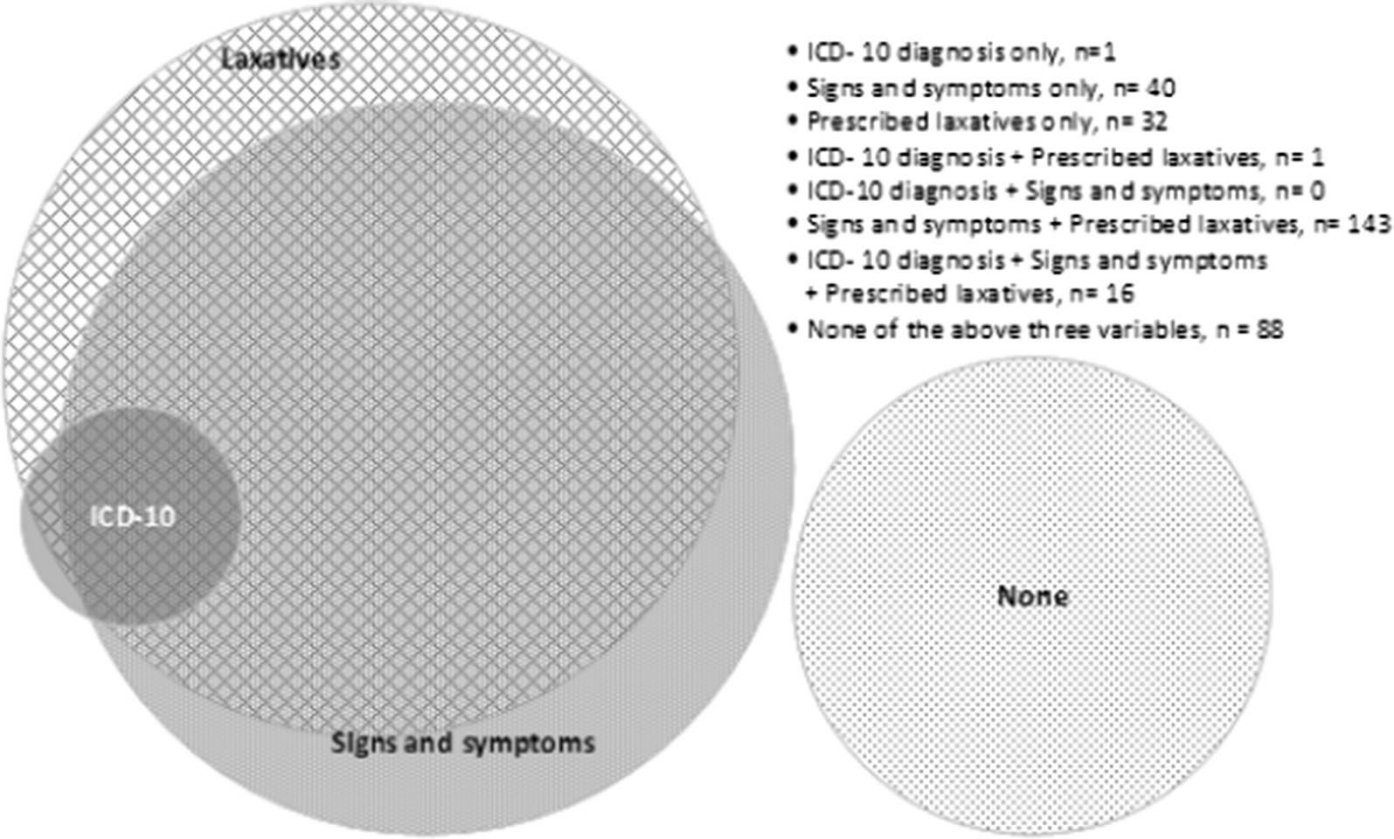
Prevalence and overlaps of ICD-10 diagnosis, signs and symptoms, and prescribed laxatives in a selected group of older people admitted to a geriatric department

The Venn diagram (Fig. 1) demonstrates how identification of constipation using the three different data sources can be seen as partly overlapping and complementary. For 16 (5%), patients data from all three data sources of identification were present in the EHR. Signs and symptoms and prescribed laxatives co-existed in the EHR for 143 (45%) patients. One (0.03%) patient had only a documented ICD-10 diagnosis documented and one (0.03%) patient had only an ICD-10 diagnosis documented. Forty (12%) patients had only signs and symptoms documented. For 32 (10%) patients, prescription of laxatives was the only documentation indicating the presence of constipation. A total of 88 (27%) patients did not have any indicators of constipation documented in the EHR.

### Factors associated with signs and symptoms of constipation

The bivariate analyses of documented signs and symptoms of constipation and the independent variables resulted in significant associations for three variables (Table 2). Patients with documented signs and symptoms of constipation were (a) at risk of developing pressure ulcers, to a greater extent than patients without (36% vs. 25%; *p* = 0.03); (b) prescribed laxatives to a greater extent than patients without (80% vs. 27%; *p* ≤ 0.001); (c) prescribed opioids to a greater extent than patients without (58% vs. 38%; *p* ≤ 0.001). In addition, patients with signs and symptoms of constipation had a longer median length of stay than patients without (10 vs. 9 days; *p* = 0.004).

The logistic regression model identified that signs and symptoms of constipation were positively associated with prescribed opioids (OR = 2.254) (Table 3). There was also a positive association with longer length of stay (OR = 1.063).

**Table 3:** Logistic regression models: associated factors with signs and symptoms and prescribed laxatives during hospitalization

Dependent variable	Independent variable	Odds ratio	95% CI	*P* value
Sign and symptoms n = 199	Age (years)	1.016	0.987–1.046	0.28
	Sex	1.272	0.745–2.173	0.38
	Female (ref) versus male			
	MNA-SF	1.308	0.726–2.357	0.12
	No risk (ref) versus risk			
	BMI	0.642	0.368–1.122	0.16
	Normal weight versus underweight			
	Norton	1.507	0.850–2.671	0.30
	No risk (ref) versus risk			
	Downton	1.424	0.728–2.785	0.63
	No risk (ref) versus risk			
	CCI	0.971	0.862–1.094	**0.00**
	Opioids	2.254	1.380–3.683	
	No (ref) versus yes			
	Anticholinergics	1.372	0.656–2.869	0.40
	No (ref) versus yes			
	Length of stay	1.063	1.000–1.129	**0.05**
Prescribed laxatives during hospitalization N = 192	Age (years)	1.030	1.000–1.061	**0.05**
	Sex	1.118	0.654–1.907	0.68
	Female (ref) versus male			
	MNA-SF	1.025	0.565–1.859	0.94
	No risk (ref) versus risk			
	BMI	0.679	0.386–1.193	0.18
	Normal weight versus underweight			
	Norton	1.452	0.817–2.578	0.20
	No risk (ref) versus risk			
	Downton	1.126	0.567–2.238	0.74
	No risk (ref) versus risk			
	CCI	0.901	0.800–1.015	0.09
	Opioids	2.154	1.316–3.524	**0.00**
	No (ref) versus yes			
	Anticholinergics	1.621	0.772–3.405	0.20
	No (ref) versus yes			
	Length of stay	1.109	1.141–1.181	**0.00**

### Factors associated with prescribed laxatives

The bivariate analyses for prescribed laxatives and the independent variables resulted in significant associations for two variables (Table 2). Patients who had been prescribed opioids were prescribed laxatives to a greater extent than those not prescribed opioids (58% vs. 39%; *p* = 0.001). Patients with prescribed laxatives had a longer length of stay compared to patients without prescribed laxatives (m = 10 vs. 8; *p* ≤ 0.001).

The logistic regression model identified that being prescribed laxatives during hospitalization was significantly associated with older age (OR = 1.030), prescribed opioids (OR = 2.154) and length of stay (OR = 1.109), this indicates that patients with prescribed laxatives were to a greater extent older, were prescribed opioids, and had a longer length of stay (Table 3).

## Discussion

The results in this study, showed that the estimated prevalence of constipation among older patients admitted to hospital differs depending on the method used for identifying the condition. There are currently no universally accepted criteria for the diagnosis of constipation in the hospital environment [31]. Based on the ICD-10 diagnosis, 6% of the patients had been diagnosed with constipation. The prevalence markedly increased to 65% when notes including signs and symptoms of constipation were analyzed. Previous research has also found levels of disagreement between the ICD-10 diagnosis and documentation of signs and symptoms related to other health conditions in EHRs [32]. It has been shown that when nursing diagnosis and medical diagnosis are combined, the results better predict risk of complications, length of stay and more [33, 34]. This might also be the case in the context of constipation. Another explanation might be that the ICD-10 diagnosis documented in the patient file where only those related to the current admission. The use of the ICD-10 diagnosis for constipation resulted in low prevalence, whereas studies measuring constipation where it is self-defined by patients tend to report much higher prevalence rates [35]. In this study, 60% of patients were prescribed laxatives, indicating more than ten times the prevalence of constipation than according to the ICD-10 diagnosis. In order to evaluate this difference, we must however have in mind that some patients received laxatives to prevent constipation, while other received laxatives to treat constipation. Our results also showed that 32 (10%) patients were prescribed laxatives, without any signs and symptoms of constipation or ICD-10 diagnosis being documented in the EHR (Fig. 1). This difference in prevalence may indicate that constipation is insufficiently diagnosed and documented in the older patient population.

The prevalence of prescribed laxatives increased from 29% at admission to 60% during hospitalization, which is also in line with previous studies and could be related to changes in the patient’s situation, such as diet, medication, immobility, loss of privacy and unfamiliar surroundings [1, 17]. Pharmacological treatment of constipation with prescribed laxatives was most common, and the use of non-pharmacological treatments was rare. A previous study has also reported that non-pharmacological treatments were rarely used [1] and the general explanation suggests that a laxative is chosen since a prompt solution to the problem might be needed [36]. Non-pharmacological treatment options could be appropriate for nurses to use in clinical practice as could practical complementary methods for nurses to use in clinical practice, however; the evidence is still inadequate regarding the effect of these interventions [37]. A systematic review evaluating the effect of non-pharmacological interventions among older people living in nursing homes concluded that making comparisons between study findings is challenging due to the differences in frequency, duration and content of the interventions [38].

Documented signs and symptoms of constipation were significantly associated in the bivariate analysis with risk of developing pressure ulcers, longer length of stay, being prescribed laxatives and opioids. Being prescribed opioids and longer length of stay remained a significant risk factor in the logistic regression model. In previous studies among older persons, risk of developing pressure ulcers and risk of falling have been identified as being associated with constipation. However, in our study this was not the case. One explanation for this difference could be related to severe mobility impairment and functional impairment among the nursing home residents under investigation [25]. Furthermore, when using prescribed laxatives as a dependent variable, age, prescribed opioids and length of stay were identified as associated factors in the regression analysis. No significant associations with constipation were detected for underlying health conditions in our study, which is in line with previous research [25]. This finding indicates that comorbid health conditions may have a lower impact on the development of constipation, when measured by the CCI. However, in contrast, a previous review reported that underlying medical conditions were one of the most common risk factors for constipation [39]. It would therefor be of importance, to prevent constipation and identify persons in risk, to explore further which specific medical conditions poses a risk for the older person.

In our study, 50% of the patients were prescribed opioids and nearly one third (31%) of those were not prescribed any laxatives. Polypharmacy is rather common among older people and many types of drugs, e.g. opioids, anticholinergics, diuretics, proton pump inhibitors, iron and calcium supplements, are associated with an increased risk of constipation especially, among older people who are often prescribed opioids [40, 41]. It is recommended that laxatives should be prescribed preventatively in patients receiving opioids, but one study reported that physicians relied on other clinicians outside their purview, such as the primary care provider, to take care of problems with constipation or waited until the patient expressed concerns during follow-up assessments [42]. Although attempts have been made to increase awareness about co-prescription of laxatives in conjunction with opioids, this has shown only a small effect in terms of outcome [43]. The NANDA international terminology system aims to ensure that the nursing process is systematically and unambiguously documented, but it is rarely used as a basis for documentation in healthcare [44]. The documentation is often inadequate, with only isolated and fragmented elements included in the EHR. This was also found in our study where outcomes were documented mostly related to information, such as the evacuation of feces, without any description of the process in connection with defecating. The Venn-diagram also demonstrates a contradictory picture of identification of constipation using ICD-diagnosis, documented signs and symptoms, and prescription of laxatives. Constipation was documented according to all three data sources in the EHRs for 5% of the patients. The majority (n = 143; 45%) of the patients with documented signs and symptoms of constipation also had a documented prescription for laxatives, but they did not have an ICD-diagnosis. One patient with an ICD-diagnosis was prescribed laxatives, but without any documented signs or symptoms of constipation. Forty (12%) patients with documentation of signs and symptoms had neither an ICD-diagnosis nor prescribed laxatives documented. Problems with documentation in EHRs can be related to structural deficiencies, poor compatibility between different systems, and the use of different vocabulary to describe the nursing process [44]. The use of a variety of terminologies can lead to misinterpretation of information between healthcare professionals and can therefore potentially jeopardize patient safety [44]. It is of great importance that the documentation is performed in a structured and standardized manner, since this can facilitate better communication between healthcare professionals and enhance the quality of care for patients. It has been previously described that healthcare professionals reported not having access to standardized valid assessment scales for identifying constipation and that this was perceived as a limitation in their clinical work. This contributes to assessments taking place in an unstructured way based on healthcare professionals’ individual experience [5].

### Strengths and limitations

Since there are no accepted criteria to define constipation, one of the strengths is that we used three different data sources to assess constipation: ICD-diagnosis, prescribed laxatives, and documented signs and symptoms of constipation. Another strength is that the sample was drawn consecutively during a whole year, which reduces the risk of seasonal variations. The third strength is that the population in the study was drawn from one of the largest geriatric departments in the region and the sample size was large. The characteristics of the sample are, to large extent, similar to a study using data from three geriatric departments in the Region [45]. However, our study also has limitations. First, EHRs were excluded if the patient had a diagnosis of dementia and this might have affected the results. Patients might have had difficulties in communicating and describing their symptoms, and healthcare professionals might have had difficulty in interpreting the signs and symptoms of constipation, and this could also confound with other symptoms such as confusion; however, only 15% were excluded. Second, the risk assessments have been assessed using valid and reliable instruments, but these have been conducted by staff who may have performed the assessment in different ways and incompletely documented the results. Third, objective measures of constipation could have added to the reliability of findings. Such measures as for example CT of colon where however not possible and would not be feasible to implement in clinical practice either.

## Conclusion

The estimated prevalence of constipation varied to a large extent depending on the data source used to identify the condition. It is noteworthy, that bowel health, which is an important subject for people’s wellbeing in daily life does not seem to be prioritized in health care, despite the existence of validated assessment scales, and guidelines regarding the management of constipation. Apparently, more needs to be done to support healthcare professionals with existing valid and reliable methods for the identification and management of constipation among older patients.

## Data Availability

The datasets generated and/or analysed during the current study are not publicly available due to the safety regulations from the hospital but are available from the corresponding author on reasonable request.

## Abbreviations

BMI: Body Mass Index
HER: Electronic health record
ICD-10: International Statistical Classification of Diseases and related Health Problems
MNA-SF: Mini Nutritional Assessment Short Form
NANDA: North American Nursing Diagnosis Association
